# High Rate of Depression among Saudi Children with Type 1 Diabetes

**DOI:** 10.3390/ijerph182111714

**Published:** 2021-11-08

**Authors:** Aqeel Alaqeel, Muna Almijmaj, Abdulaziz Almushaigeh, Yasser Aldakheel, Raghad Almesned, Husam Al Ahmadi

**Affiliations:** 1 Department of Pediatrics, College of Medicine, Qassim University, Qassim 51452, Saudi Arabia; 2 Department of Pediatrics, Dr Suliman Al Habib Hospital, Riydah 13212, Saudi Arabia; m.ab.almijmaj@gmail.com; 3 Emergency Medicine Department, Riyadh Al khabra Hospital, Qassim 52714, Saudi Arabia; Abdulazizmoshaigeh@gmail.com; 4 Department of Pediatrics, King Fahad Medical City, Riydah 12231, Saudi Arabia; sul3saleh@gmail.com; 5 Family Medicine Academy, Qassim 52211, Saudi Arabia; Raghadalmisned@gmail.com; 6 Maternity & Children Hospital, Al Qassim 52384, Saudi Arabia; husamhmh@hotmail.com

**Keywords:** type 1 diabetes, children’s depression inventory, depression, children, Saudi Arabia

## Abstract

Saudi Arabia ranks among the top ten in type 1 diabetes (T1D) prevalence. The psychological burden, including depression, among T1D children, affects short-term and long-term outcomes. In Saudi Arabia, studies on depression among T1D children are limited. We determined the prevalence of depression among T1D children and adolescents in the Saudi Arabia-Qassim region and investigated risk factors for depressive symptoms. This quantitative cross-sectional study was conducted among T1D children and adolescents in the outpatient clinic of Maternity and Children Hospital, Buraydah, Saudi Arabia, between October 2020 and April 2021. Using a validated questionnaire translated into Arabic, we interviewed patients during clinic appointment. Questionnaires on sociodemographic characteristics, clinical data, and Clinical Depression Inventory scale were used to measure depression. There were 148 T1D respondents (children: 58.1%; adolescents: 41.9%). More than half were females (53.4%), with most Saudis (94.6%). Depression prevalence among children and adolescents was 27%. Mild, moderate, and severe depression occurred in 80%, 12.5%, and 7.5% of depressed patients, respectively. Factors significant for depression were female sex (*p* = 0.014), uncontrolled HbA1c level (*p* = 0.037), and longer diabetes duration (*p* = 0.013). Depression among children and adolescents was more prevalent in this study than in previous reports. Early detection of depression will improve diabetes control and quality of life.

## 1. Introduction

It is well known that diabetes mellitus (DM) patients have two to three times higher risk of developing psychological disorders, mainly depression, than healthy individuals [1]. There is a considerable amount of published literature on how DM could increase the risk of depression. One of the biological theories suggests that severe glucose fluctuations may cause changes in the central nervous system that are responsible for cognition and mood [2]. Furthermore, it is well known that diabetes patients have higher levels of inflammatory markers, which may contribute to the development of depressive symptoms [3].

Compared to other chronic disorders where major decisions fall on the medical team, the obligation of diabetes with regard to its management is fundamentally dependent on patients and their families. The burden of diabetes is represented by its demand for a precise daily routine, which includes day-to-day monitoring of blood glucose levels, nutrition, and insulin dosing [4]. Diabetes imposes overwhelming stress in youths with well controlled glucose levels, as they take steady care and are in constant dread of hypoglycemia [5]. In addition, these patients are continuously expected to take action rapidly in case of a hypo/hyperglycemic event [4,5,6]. A prior report documented an increased risk of depression even in patients treated with advanced technology, including insulin pumps, who have good glycemic control. Thus, all these factors can lead to burn-out in patients with well-controlled glycemic levels [6].

It is crucial to diagnose and treat depression associated with T1D, as it may increase the rate of acute complications, worsen glycemic control, and reduce quality of life [1,7,8]. Lastly, it is worth mentioning that adolescents with diabetes tend to have a higher risk of suicidal ideation than healthy adolescents [9]. 

Prior studies have linked depression to DM duration, poor glycemic control, sex, and BG measurement frequency in children and adolescents with T1D [10,11,12]. However, systemic reviews showed either slightly increased depression or uncertain results as to whether diabetes increased the risk of depressive symptoms [4,13].

We intended to discover whether depression is highly prevalent among children and adolescents with T1D in our community in Saudi Arabia so that an early screening program with appropriate intervention could be suggested for management.

There are limited data on the prevalence of depression in children and adolescents with T1D in Saudi Arabia. This evaluation is very important in this age group, especially in view of the absence of psychological assessment for this population. We aimed to determine the prevalence of depression among T1D children and adolescents in the Qassim region and investigate the risk factors contributing to the development of depressive symptoms.

## 2. Materials and Methods

### 2.1. Study Design and Setting

This cross-sectional study is based on the Quantities approach performed among children and adolescents with T1D in the outpatient clinic of pediatric and endocrinology departments in Maternity and Children Hospital Buraydah, Saudi Arabia, between October 2020 and April 2021. Buraydah is the capital city of the Qassim region in Saudi Arabia, with a population of 689,318.

### 2.2. Patients 

Patients aged 8–16 years, including both sexes, who were diagnosed with T1D, receiving care for a minimum of 6 months, and could read Arabic independently, with adequate intellectual capacity, were eligible for inclusion into the study. The following were excluded from the study: patients with type 2 diabetes, major psychiatric disorder, patients who have other associated disorder or complications related to DM. In addition, patients who were admitted in the past 3 months, or who refused to provide consent were excluded from the study. 

The authors reviewed all participants’ charts and excluded patients who did not meet entry criteria. They then interviewed the patients at the time of the participants’ clinic appointment. We used the Arabic version of the Children’s Depression Inventory (CDI) questionnaire for the interviews [14]. After obtaining consent from the patients’ parents, during the routine follow-up visits, we gathered the necessary data and socio-demographic data, including patient sex, age, and nationality, as well as clinical data, including patient’s HbA1c, DM duration, and methods of insulin administration. In addition, body mass index (BMI) was calculated as weight in kg divided by height in m^2^. BMI-for age percentiles were expressed as underweight (BMI < 5th percentile), normal weight (BMI ≥ 5th and <85th percentile), overweight (BMI ≥ 85th and <95th percentile), and obese (BMI ≥ 95th percentile). History of any diabetic ketoacidosis (DKA) events, except at diagnosis and regardless of the number of events, was taken from medical records. DKA was defined based on ISPAD criteria [15]. The patient then completed the Arabic translated version of the (CDI) questionnaire independently using a self-administered data collection technique.

The CDI questionnaire is often used in clinical studies because of its high reliability coefficient (Cronbach’s a = 0.81–0.89). The questionnaire comprises 27 items with three answers for each item. Each answer was scored from 0 to 2, and the patient chose the answer depending on the feelings experienced in the past two weeks. The total score ranged from 0 to 54. The Institutional Review Board of the Qassim Region approved the study.

### 2.3. Scoring

Many countries followed a score of 13 as the cut-off point for clinical depression symptoms. However, the validated Arabic version recommends a score of 15 or higher. Higher scores reflect greater symptomatology subscales testing different depressive symptoms: negative mood, loss of interest in daily activities, loss of energy, negative self-esteem, and interpersonal problems [14,16]. Details of Arabic CDI score is attached in the Supplementary Materials.

### 2.4. Statistical Analysis

The Statistical Package for the Social Sciences (SPSS), version 26 (IBM Corporation, Armonk, NY, USA) was used to analyze the data. Descriptive statistics are presented as numbers and percentages. A *p*-value cut-off point of 0.05, at a 95% confidence interval (CI), was used to determine statistical significance. In the analyses, we measured the association between sociodemographic characteristics and depression using the chi-square test (categorical variables) and independent-samples *t*-test (continuous variables). Significant results were then inputted into a multivariate regression model to ascertain the effect of depression from the selected demographic characteristics of the patients, where the adjusted odds ratio (OR) and 95% CI, were also being reported.

## 3. Results

This study included a total of 148 children. Of them, approximately 60% were children (8–12 years), and more than half of the children were girls (53.4%); nearly all children were Saudis (94.6%). The sociodemographic details of the patients are presented in Table 1. Uncontrolled HbA1c level (>7.5%) was noted in 78.4% of the patients. Most children had normal BMI (63.5%), whereas the others were overweight (17.6%) or obese (10.8%). Furthermore, more than one-third (34.5%) of the patients had a diabetes duration of more than 5 years. The most common method of insulin administration was multiple daily injections (81.7%). In addition, 46.6% of the children had episodes of DKA.

**Figure 1:**
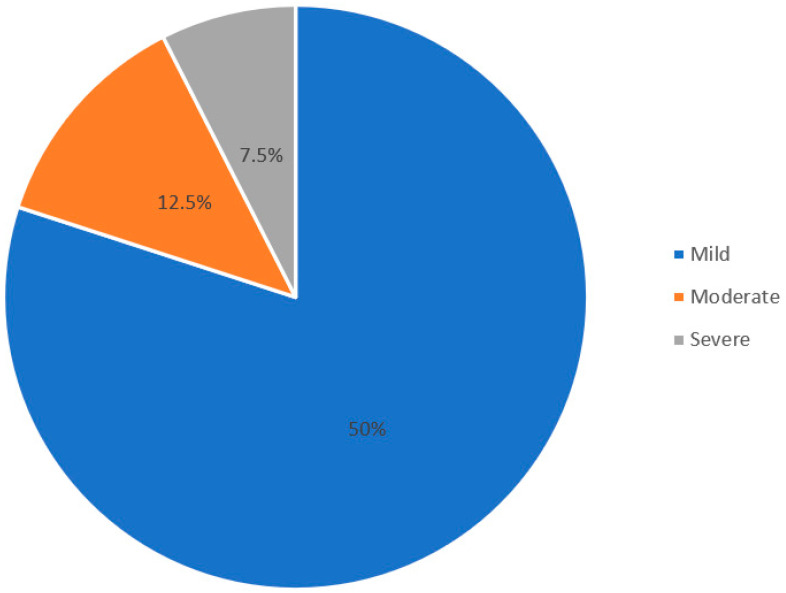
Proportion of patients based on severity of depression.

Table 2 shows the prevalence of depression. Depressive symptoms were detected in 27% of patients. Of them, 80% were classified as having mild depression, 12.5% as having moderate depression, and 7.5% as having severe depression (Figure 1).

We used the chi-square test and the independent-samples *t*-test to evaluate the relationship between depression among the different socio-demographics and the incidence of DKA. The results showed that the prevalence of depression was more common among females (*p* = 0.014), those with poor glycemic control (*p* = 0.037), and those with DM duration of 5 years or more (*p* = 0.013). However, age group in years, nationality, BMI, method of insulin administration, and history of DKA were not significant parameters when compared with depression (*p* > 0.05) (Table 3).

Multivariate regression analysis was subsequently performed to determine the effect of depression on the selected socio-demographic characteristics of the children. The results are shown in Table 4. The results revealed that the risk of depression among female subjects was four times higher than that among male subjects (adjusted OR [AOR] = 4.552; 95% CI = 1.805–11.476; *p* = 0.001). Patients with uncontrolled HbA1c levels were seven times more likely to have depression (AOR = 7.122; 95% CI = 1.927–26.320; *p* = 0.003). Furthermore, patients with a DM duration of 6 months to 1 year were sevenfold more likely to have depression (AOR = 7.620; 95% CI = 2.145–27.066; *p* = 0.002), while the likelihood ratio for patients with a DM duration of 5 years or more was 4.8 times higher (AOR = 4.820; 95% CI = 1.073–21.653; *p* = 0.040).

## 4. Discussion

T1D is one of the most chronic diseases in children and is highly prevalent in Saudi Arabia [17,18,19]. It requires lifelong management, which can contribute to and affect the patient’s life from many aspects, mainly in terms of psychological burden and quality of life [20,21]. In the current study, we measured the prevalence of depression among T1D children and adolescents and the association between sociodemographic characteristics and depression. Regarding the reported data on healthy children from different countries, including Italy, Spain, Sweden, and Cyprus, wherein the CDI score was used, the depression rates were 8–15% [21].

On the other hand, based on results of McGrade and Hood [12], 23% of 144 adolescents with T1D scored above the clinical cutoff of depression (CDI score ≥ 13), our results are consistent with this finding, as it shows that approximately a quarter (27%) of children with T1D had depressive symptoms (CDI score ≥ 15), which was also in line with the findings of another study conducted in the USA and Poland [10,22].

In this study, sex was one of the factors associated with depression, and based on univariate and multivariate analyses, depression was more prevalent in females and was predicted to be 4.5 times higher than that of their male counterparts. These results corroborate the findings of previous reports who found that depression was more likely to occur in females, mainly in the adolescence age group [1,10,11,22]. This finding may be explained by the fact that healthy adolescent females in Saudi Arabia tend to have higher rates of depression and poor quality of life owing to increased worries, less satisfaction of life, and poor health perception; they are also known to skip insulin doses, which contributes to disturbed metabolic control [20,23,24]. However, some studies did not predict sex-based differences in depression among youth participants, but they found significant differences in depression levels across the age groups, suggesting that adolescents had the highest depression rates [25,26]. In our study, age was not a relevant factor for depression, which did not coincide with the findings of previous reports. The cut-off age in pediatric diabetes centers of Saudi Arabia is 14 years, and some extend it to 16 years, which explains why there are fewer adolescent patients, and thus, we cannot find an increased depression rate in this age group.

The duration of T1D in children is an important factor that causes depressive symptoms; our results showed that more than half of the children with depression had T1D for more than 5 years. Studies conducted in Egypt, Poland and the United States have revealed the same relationship [1,6,11,27]. This finding may be explained by prolonged exposure to the stresses of T1D management and the higher rate of acute complications. On the contrary, most previous studies have focused on the long-term risk of depressive symptoms in diabetes patients. However, we found that DM in the first year of diagnosis carries a high risk of depression, with a *p*-value of 0.002, which emphasizes the importance of the initial duration and psychological burden of diabetes early on and the importance of early psychological evaluation and psychological support sessions to help diabetes patients cope with the burden of diabetes.

Another reported association with depression was lower blood glucose monitoring frequency that lead to poorer glycemic control and eventually higher risk of depression [12,28]. Most of our patients are on continuous glucose monitoring (Freestyle libre) so we did not include the blood glucose checking frequency in the variables.

Most of the previous reports documented that T1D children and adolescents have higher risk of depressive symptoms [4,11,12].

Consistently, in our study, poor control was associated with an increased rate of depression. On the contrary, Sendela et al. [25] documented that there were no differences in glycated hemoglobin levels in school-aged children with diabetes with and without depression. Some investigators have claimed that depression may contribute to poor glycemic control; however, the converse may also occur [10].

Increased DKA episodes have been linked to poor quality of life [20,21]; however, in our study, the depression scale was not increased in patients with DKA. This may be attributed to the study methodology, such as self-reported, small sample size, and finally, we did not include DKA at diagnosis or number of events which could be a limitation of this study. Moreover, we were unable to determine whether the insulin regimen was related to depressive symptoms because most of the patients in our study were on a multiple daily injection regimen and few patients were on insulin pump therapy. A previous study conducted in Poland documented an increased rate of depression in T1D adolescents who use insulin pump regardless of glycemic control [6].

The American Diabetes Association and ISPAD recommend screening psychological disorders including depression at diagnosis and at planned intervals for depression as part of routine best practice [29]. Furthermore, we emphasize the recommendation of multidisciplinary pediatric diabetes teams to provide early detection and adequate clinical intervention plans for those identified through screening for depressive symptoms [21,30].

Additionally, a process should be established to discuss symptoms of depression with caregivers and take appropriate measures, including emergency procedures for those with suicidal ideation. A literature review on family support for children with diabetes showed that greater family cohesion and organization are associated with better psychosocial outcomes [31].

Lastly, it is important to encourage physical exercise, regular sleep hours, play therapy, cognitive behavioral therapy, peer support, family support group therapy and most importantly psychological referral, who will make the decision regarding need to start anti-depressant medication. All of this can help improve the mental health of diabetic children and help them cope with their depression [32,33].

There were certain limitations of the study. First, the study was conducted at a single center, and there was no control group or reference to population norms to compare the study group. Second, like any self-reported questionnaire, the CDI tool may lead to self-report bias. For example, some of studied participants may under report their symptoms in order to minimize their problem or may exaggerate their response. Some may misunderstand the meaning of certain questions as most of our participants were children. In order to reduce self-report bias, we suggested that any child with a positive CDI score was re-evaluated by a specialized psychiatrist. Finally, we could have had a larger sample size but the entry criteria and the telehealth system used (due to COVID-19 precautions) limited our sample size. Further studies overcoming these limitations are required.

## 5. Conclusions

Depression among children and adolescents is widely prevalent in this study compared with that in previous studies. Female sex, poorly glycemic control, and longer diabetes duration were significant factors associated with depression. Depressive symptoms can barely be recognized among children, and we ascertain that early detection of depression will lead to better diabetes control, eventually leading to better quality of life among children with T1D. Thus, early recognition of the signs of depression is important to provide the necessary therapy among the youth with diabetes.

## Figures and Tables

**Table 1:** Sociodemographic profiles of children with type 1 diabetes (*n* = 148).

Study Variables	*N* (%)
Age group	
8–12 years	86 (58.1%)
13–16 years	62 (41.9%)
Gender	
Male	69 (46.6%)
Female	79 (53.4%)
Nationality	
Saudi	140 (94.6%)
Non-Saudi	08 (05.4%)
HbA1c	
≤7.5%	32 (21.6%)
>7.5%	116 (78.4%)
BMI level	
Underweight (<5th)	12 (08.1%)
Normal (5th–<85th)	94 (63.5%)
Overweight (85th–<95th)	26 (17.6%)
Obese (>95th)	16 (10.8%)
DM Duration	
6 months–1 year	39 (26.4%)
1–2 years	24 (16.2%)
2–3 years	19 (12.8%)
3–4 years	15 (10.1%)
>4 years	51 (34.5%)
Method of insulin administration	
Multiple daily injections without carbohydrate calculation	102 (68.9%)
Multiple daily injections with carbohydrate calculation	19 (12.8%)
Insulin pump	02 (01.4%)
Premixed insulin	25 (16.9%)
Episodes of DKA in the past year?	
Never	79 (53.4%)
One time	40 (27.0%)
Twice times	14 (09.5%)
≥Three times	15 (10.1%)

BMI, body mass index; DKA, diabetes ketoacidosis; DM, diabetes mellitus.

**Table 2:** Prevalence of depression and its severity (*n* = 148).

Depression	*N* (%)
Level of depression	
Depressed	40 (27.0%)
Not depressed	108 (73.0%)
Severity of depression (*n* = 40)	
Mild	32 (80.0%)
Moderate	05 (12.5%)
Severe	03 (07.5%)

**Table 3:** Relationship of depression level and socio-demographic profiles with hypoglycemic, DKA events, and measurement of blood glucose levels per day (*n* = 148).

Factor	Depressed *N* (%) (*n* = 40)	Not Depressed *N* (%) (*n* = 108)	*p*-Value ^§^
Age group			
8–12 years	20 (50.0%)	66 (61.1%)	0.224
13–16 years	20 (50.0%)	42 (38.9%)	
Gender			
Male	12 (30.0%)	57 (52.8%)	0.014 **
Female	28 (70.0%)	51 (47.2%)	
Nationality			
Saudi	38 (95.0%)	102 (94.4%)	0.894
Non-Saudi	02 (05.0%)	06 (05.6%)	
HbA1c			
≤7.5%	04 (10.0%)	28 (25.9%)	0.037 **
>7.5%	36 (90.0%)	80 (74.1%)	
BMI level			
Underweight	04 (10.0%)	08 (07.4%)	0.593
Normal	27 (67.5%)	67 (62.0%)	
Overweight/Obese	09 (22.5%)	33 (30.6%)	
DM Duration			
6 months–1 year	04 (10.0%)	35 (32.4%)	0.013 **
1–2 years	06 (15.0%)	18 (16.7%)	
2–3 years	05 (12.5%)	14 (13.0%)	
3–4 years	03 (07.5%)	12 (11.1%)	
>4 years	22 (55.0%)	29 (26.9%)	
Method of insulin administration			
Multiple daily injections without carbohydrate calculation	32 (80.0%)	70 (64.8%)	0.234
Multiple daily injections with carbohydrate calculation	03 (07.5%)	16 (14.8%)	
Insulin pump	01 (02.5%)	01 (0.90%)	
Premixed insulin	04 (10.0%)	21 (19.4%)	
History of DKA event			
Yes	19 (47.5%)	50 (46.3%)	0.896
No	21 (52.5%)	58 (53.7%)	

BMI, body mass index; DKA, diabetes ketoacidosis; DM, diabetes mellitus; ^§^ *p*-value was calculated using the chi-square test. *p*-value was calculated using the independent-samples *t*-test. ** Significance level at *p* < 0.05.

**Table 4:** Multivariate regression analysis to determine the effect of depression on the selected socio-demographic characteristics of patients (*n* = 148).

Factor	AOR	95% CI	*p*-Value
Gender			
Male	Ref		
Female	4.552	1.805–11.476	0.001 **
HbA1c			
≤7.5%	Ref		
>7.5%	7.122	1.927–26.320	0.003 **
DM Duration			
6 months–1 year	Ref		
1–2 years	7.620	2.145–27.066	0.002 **
2–3 years	1.895	0.557–6.448	0.306
3–4 years	1.469	0.412–5.238	0.553
>4 years	4.820	1.073–21.653	0.040 **

AOR, adjusted odds ratio; CI, confidence interval; Ref, reference; DM, diabetes duration. ** Significance level at *p* < 0.05.

## Data Availability

The data presented in this study are not publicly available due to privacy policy but are available on request from the corresponding author.

## Supplementary Materials

The following are available online at https://www.mdpi.com/article/10.3390/ijerph182111714/s1, Table S1: Classification of depression-Male, Table S2: Classification of depression-Female.
