# Supplementary material for: High Rate of Depression among Saudi Children with Type 1 Diabetes
Source: Int J Environ Res Public Health. 2021 Nov 8;18(21):11714. doi: 10.3390/ijerph182111714 (PMC8583043; doi:10.3390/ijerph182111714)
Supplement: Supplementary file 1 [file ijerph-18-11714-s001.zip › ijerph-1445671-supplementary.pdf]

Classification of depression:

Male:

age	Non	mild	moderate	sever
7 years+6months to 10 years+ 5 months	0-14	15-22	23-29	30 or more
10 years +6 months to 13years+ 5 months	0-14	15-21	22-27	28 or more
13 years+ 6months to 16 years+ 5 months	0-14	15-21	22-28	29 or more

Female:

age	Non	mild	moderate	sever
7 years+6months to 10 years+ 5 months	0-14	15-22	23-29	30 or more
10 years +6 months to 13years+ 5 months	0-16	17-23	24-30	31 or more
13 years+ 6months to 16 years+ 5 months	0-18	19-24	25-30	31or more
